# Correlation Analysis of Pyroptosis-Related Genes CASP1, NLRP3, AIM2, and NLRP1 With Lung Adenocarcinoma

**DOI:** 10.1155/ijog/8282590

**Published:** 2025-02-22

**Authors:** Lingling Zhu, Yongqian Zhang, Xiaojing Chen, Yuehang Li, Haiqiao Pan, Yuan Wang, Ning Chen, Yajing Wu, Yishuai Li, Min Zhao

**Affiliations:** ^1^Department of Oncology, The First Hospital of Hebei Medical University, Shijiazhuang, Hebei, China; ^2^Graduate School, Hebei Medical University, Shijiazhuang, Hebei, China; ^3^Department of Respiratory and Critical Care Medicine Ward 1, Handan Central Hospital, Handan, Hebei, China; ^4^Graduate School, Chengde Medical University, Chengde, Hebei, China; ^5^Department of Respiratory, Hebei Chest Hospital, Shijiazhuang, Hebei, China; ^6^Department of Pathology, The First Hospital of Hebei Medical University, Shijiazhuang, Hebei, China; ^7^Department of Radiotherapy, The Fourth Hospital of Hebei Medical University, Shijiazhuang, Hebei, China; ^8^Department of Thoracic Surgery, Hebei Chest Hospital, Shijiazhuang, Hebei, China

**Keywords:** adenocarcinoma of the lung, diagnose, immunity, prognosis, pyroptosis

## Abstract

**Purpose:** This study is aimed at exploring the role of pyroptosis-related genes in the development, immune infiltration, and clinical features of lung adenocarcinoma.

**Method:** Pyroptosis-related genes were searched using online databases, including MSigDB, Gene, and GeneCards. We explored pyroptosis-related gene expression patterns in lung adenocarcinoma using the UALCAN database. Functional enrichment analysis of pyroptosis-related genes in lung adenocarcinoma was performed using the Metascape database. A protein–protein interaction network was constructed using the STRING database, and the outcomes were visualized using Cytoscape. The top five core genes were screened utilizing the MCC algorithm with its cytoHubba plugin. The correlation between immune cell infiltration, diagnosis, and prognosis of core genes in lung adenocarcinoma was explored using the TIMER 2.0, TCGA, and Kaplan–Meier plotter databases. A nomogram was constructed to predict the survival of patients with lung adenocarcinoma using Cox regression analysis, and its clinical value was validated. Samples of paraffin-embedded lung adenocarcinoma tissues were collected and subjected to immunohistochemical tests to verify the expression of core genes in lung adenocarcinoma and adjacent tissues.

**Results:** Overall, 202 genes related to pyroptosis were identified, with 67 upregulated and 60 downregulated in lung adenocarcinomas. The top five core genes—namely, CASP1 (caspase1), PYCARD (PYD and CARD domain-containing protein), NLRP3 (NOD-like receptor protein 3), AIM2 (absent in melanoma 2), and NLRP1 (NOD-like receptor protein 1)—related to lung adenocarcinoma pyroptosis were selected. The correlation analysis of immune cell infiltration showed that CASP1, NLRP3, and AIM2, which showed that pyroptosis was involved in the infiltration of immune cells in the tumor microenvironment and NLRP1 exhibited high diagnostic efficacy, while PYCARD demonstrated poor diagnostic efficacy. High expression of CASP1, NLRP3, and NLRP1 correlated with a better prognosis (*p* < 0.05), while elevated AIM2 expression was associated with a poor prognosis (*p* < 0.05). However, PYCARD exhibited no significant correlation with prognosis (*p* > 0.05). The immunohistochemistry results showed that positive rates of CASP1, NLRP3, AIM2, and NLRP1 were 20%, 15%, 70%, and 10%, respectively, while in adjacent tissues, the positive rates were 60%, 60%, 20%, and70%, indicating high expression of AIM2 and low expression of CASP1, NLRP3, and NLRP1 in lung adenocarcinoma.

**Conclusion:** CASP1, NLRP3, AIM2, and NLRP1 are core pyroptotic genes in lung adenocarcinoma and exhibit a strong correlation with immune cell infiltration, diagnosis, and prognosis of this condition. These genes may be useful in the clinical diagnosis and treatment of patients with lung adenocarcinoma.

## 1. Introduction

Lung cancer stands as one of the most prevalent malignancies worldwide, ranking foremost in cancer-related mortality, with a significant share of 18.0% [1]. Lung adenocarcinoma is the most common histological type of lung cancer. Despite the fact that advancements in treatment modalities such as surgery, radiotherapy, chemotherapy, targeted therapy, and immunotherapy had significantly improved patients' survival, the majority of patients are diagnosed at an advanced stage, missing the opportunity for curative treatment. Therefore, enhancing therapeutic efficacy in patients with lung adenocarcinoma is crucial. Investigating genetic or molecular targets holds significance in advancing early diagnosis and predicting prognosis.

Pyroptosis is a newly discovered form of programmed cell death characterized by an atypical process involving an inflammatory reaction. This process mainly relies on the cleavage activity of the cysteine aspartate protease (caspase (CASP)) family, which activates gasdermin proteins. These activate gasdermin protein migration to the cell membrane, causing pore formation and cell death. Simultaneously, this process promotes the release of inflammatory molecules into the extracellular environment, eliciting inflammation and triggering immune response [2]. Pyroptosis has garnered increasing attention as a novel type of programmed cell death. Besides identifying the role of pyroptosis in conditions such as microbial infection, atherosclerosis, osteoarthritis, and diabetic nephropathy, its close association with tumors has also been established. Several studies have demonstrated the association of pyroptosis-related molecules—such as GSDMD, CASP1, melanoma deficiency factor 2 (absent in melanoma 2 (AIM2)), NOD-like receptor protein (NLRP1), NOD-like receptor protein 3 (NLRP3), NLR family-containing CARD structural protein 3 (NLRC3), and NLR family CARD domain-containing protein 4 (NLRC4)—with various tumors. In gastric cancer studies, a decreased GSDMD expression has been observed, potentially promoting the proliferation of gastric cancer cells [3]. Barrett esophageal adenocarcinoma often originates from precancerous lesions in the esophagus. One contributing factor involves the activation of IL-1 *β* and IL-18 secretion facilitated by CASP1 [4]. Additionally, in hepatocellular carcinoma, AIM2 plays an important role in pyroptosis by regulating the mTOR signaling pathway [5]. Elevated gasdermin protein expression has been observed in serous ovarian cancer [6]. Reduced expression levels of the pyroptotic inflammasome-related components (NLRP1, NLRP3, NLRC3, NLRC4, and AIM2) have been observed in a murine model of colorectal cancer [7]. An imbalance in pyroptosis-related molecular expression affects the immune microenvironment in bladder cancer [8]. Pyroptosis-related pathways are involved in tumor immune responses, including the inflammasome, caspase, and gasdermin families mentioned above. Pyroptosis is a cell death pathway that plays an important role in immune regulation of cancer. Some in vivo and in vitro tests have shown that pyroptosis has a great impact on anticancer immunity, which means that the study of pyroptosis can promote the application of immunotherapy. Studies on the correlation between pyroptosis and malignancy can be found. This being the case, more research is needed to understand the mechanism of the relationship between pyroptosis and clinical treatment [9]. Pyroptosis has emerged as a new avenue in tumor research.

Bioinformatics is an interdisciplinary discipline that can use the genomic information of lung cancer cells to identify key genetic mutations associated with lung cancer through bioinformatics tools. By analyzing the data of a large number of lung cancer patients, the biomarkers related to the occurrence, development, prognosis, and treatment response of lung cancer were identified, and these markers could be used for the early diagnosis, prognosis assessment, and treatment guidance of lung cancer. Although bioinformatics plays a huge role in biomedical research, it also has some limitations, such as the lack of standardization of data generated by different experimental platforms and different laboratories, leading to difficulties in data integration and analysis. And bioinformatics analysis often requires a lot of computational resources, especially when working with large datasets such as high-throughput sequencing data. This can be computationally expensive, limiting some research [10].

Pyroptosis has been linked to the development of several tumors [11]. However, its relationship with lung adenocarcinoma remains poorly understood. Therefore, this study is aimed at exploring the role of pyroptosis-related genes in the development, immune infiltration, and clinical features of lung adenocarcinoma using bioinformatics analyses. Immunohistochemistry was used to verify the expression pattern of core pyroptotic genes, known for their clinical relevance to lung adenocarcinoma and normal lung tissues, thereby providing a new idea for the clinical diagnosis and treatment of lung cancer.

## 2. Materials and Methods

### 2.1. Acquisition of Pyroptosis-Related Gene Databases in Lung Adenocarcinoma

**Figure 1:**
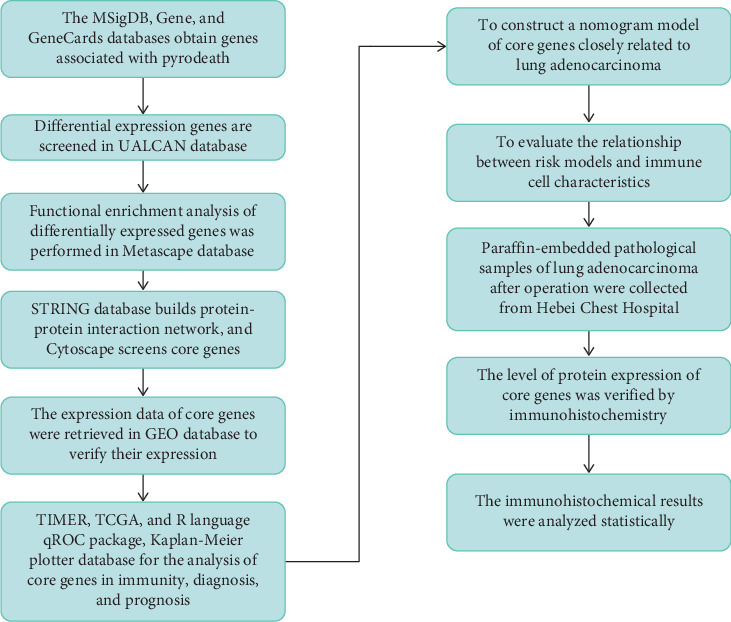
Flowchart.

In this study, the core genes of lung adenocarcinoma were analyzed using bioinformatics.  Figure 1 shows the detailed study procedures.

Using the MSigDB (https://www.gsea-msigdb.org/gsea/msigdb) [12], Gene (https://www.ncbi.nlm.nih.gov/gene) [13], and GeneCards (https://www.genecards.org/) [14] databases to obtain genes related to apoptosis, the union of results from these three databases is taken, and the expression of apoptosis-related genes in lung adenocarcinoma is analyzed in the UALCAN database [15]. The expression of pyroptosis-related genes in lung adenocarcinoma was analyzed in medium. Among these databases, the MSigDB contains annotated gene sets associated with biochemical pathways, signaling cascades, expression profiles in research publications, and various biological concepts. The Gene database can be found in the National Center for Biotechnology Information (NCBI). The GeneCards database constitutes a comprehensive and authoritative compilation of annotative information regarding human genes. The UALCAN database (https://ualcan.path.uab.edu/) is a web-based tool for analyzing and extracting pertinent cancer data in The Cancer Genome Atlas (TCGA) database (https://www.cancer.gov/ccg/research/genome-sequencing/tcga). It facilitates gene expression profiling, methylation analysis, and the analysis of differentially expressed genes (DEGs) in normal tissues.

### 2.2. Enrichment Analysis of Pyroptosis-Related Genes in Lung Adenocarcinoma

The objective of the gene enrichment analysis was to enrich genes across various pathways and explore the functional roles of pyroptosis-related genes in lung adenocarcinoma-associated. The primary functions of the Metascape database (https://metascape.org) [16] encompass gene annotation, gene function, pathway enrichment analysis, and protein–protein interaction (PPI) relationship inference. The Metascape database was employed to conduct Gene Ontology (GO) and Kyoto Encyclopedia of Genes and Genomes (KEGG). The GO enrichment mainly involved biological processes (BPs), cell components (CCs), and molecular function. Genes within the functional domain of lung adenocarcinoma cells were entered and subjected to customized analysis in *Homo sapiens*. The screening conditions were evaluated using the default parameters (min overlap: 3, *p* value cutoff: 0.01, min enrichment: 1.5).

### 2.3. Acquisition of Pyroptotic Core Genes in Lung Adenocarcinoma

The PPI network was constructed using the STRING database (https://www.string-db.org) [17]. Subsequently, the results were imported into the Cytoscape software [18] for visualization using the cytoHubba plugin [19]. The MCC algorithm was utilized to select the top five core genes. The STRING database is a biological database and web resource of known proteins and predicted PPI relationships. The parameters set for the STRING database included network type: full STRING network, required score: highest confidence (0.900), and FDR stringency: medium (5%). Cytoscape is a software program primarily designed for open-source network visualization. It was used to analyze PPI visualization and computation of core genes.

### 2.4. Validation of Core Gene Expression

The lung adenocarcinoma dataset was retrieved to verify the reliability of core gene expression obtained from the GEO database (https://www.ncbi.nlm.nih.gov/geo/) [20]. Subsequently, the expression data of the core genes were acquired utilizing the jvenn online tool [21]. Furthermore, the differential expression of these core genes was visualized in lung adenocarcinoma. The Gene Expression Omnibus (GEO) database is a public repository for functional genomic data. The jvenn tool functions as a simple JavaScript plugin offering multiple functions.

### 2.5. Association Analysis of Core Genes, Diagnosis, Prognosis, and Immunity

To explore the correlation between immune cell infiltration, diagnosis, and prognosis of core genes in lung adenocarcinoma, the TIMER 2.0 [22], TCGA [23], and Kaplan–Meier plotter databases [24] were utilized for analysis, respectively. The TIMER 2.0 database (http://timer.cistrome.org/) is a comprehensive resource for systematically analyzing immune cell infiltration in different cancer types. This database was used to explore the relationship between core gene expression and immune cell infiltration in lung adenocarcinoma in this study. TCGA (https://www.cancer.gov/ccg/research/genome-sequencing/tcga) collects clinicopathological data from various tumors across 33 cancer types. From TCGA, RNA-seq data from 535 lung adenocarcinoma projects were retrieved, with 59 specifically matched to adjacent tissues. The downloaded data were initially in Level 3 FPKM format and were later converted to TPM for subsequent analyses. Duplicate samples were eliminated, and data containing only clinical information were retained for further analyses. Subject operating characteristic (ROC) curves were generated using the pROC package to analyze the diagnostic efficacy of core genes in lung adenocarcinoma. Consequently, the results were visualized using the ggplot2 software package. The Kaplan–Meier plotter database (https://kmplot.com/analysis/) collects the survival data sourced from databases such as GEO and TCGA. This website was utilized for prognostic analysis of core genes in lung adenocarcinoma. The parameters were configured as follows: patients were split using the “auto select best cutoff” settings, survival set to “OS,” probe set options set to “only JetSet best probe set,” and histology set to “adenocarcinoma.”

### 2.6. Prognostic Nomogram Model Construction for Patients With Lung Adenocarcinoma

The nomogram figure represents the condition of each variable in the multivariate regression model based on multifactor regression analysis. Furthermore, the total score is calculated to predict the probability of events [25]. A nomogram was constructed using Cox regression in the R language to develop a clinical quantitative tool capable of predicting 1-, 3-, or 5-year OS in patients with lung adenocarcinoma. Using TNM stage, pathological stage, and expression level of core genes of lung adenocarcinoma, we construct a nomogram based on Cox regression using R language and establish a calibration chart to test the prediction efficiency of the nomogram model. This process is performed using the Survival Package and RMS Package.

### 2.7. Assessment of Tumor Immune Cell Infiltration

To evaluate the relationship between the risk model and immune cell characteristics, immune infiltration data was computed for the samples in the lung adenocarcinoma dataset from TCGA project. Subsequently, the relationship between risk score values and immune infiltrating cells was analyzed utilizing the ssGSEA algorithm and Spearman correlation analysis. The significance threshold was set at *p* < 0.05. This process was performed using the R ggplot2 software package.

### 2.8. Source of the Specimens

Immunohistochemistry was conducted on 20 paraffin-embedded pathological samples, with 10 cases of lung adenocarcinoma from thoracic surgical excision specifically selected from January 2020 to December 2021. None of the patients had undergone chemotherapy, radiotherapy, targeted therapy, or treatment for other lung diseases, tumors, diabetes, or rheumatoid arthritis before undergoing radical surgery. The Hebei Chest Hospital Ethics Committee approved all wax block tissue specimens used in this study (Batch Number 2022025).

### 2.9. Immunohistochemistry

#### 2.9.1. Main Reagent

The reagents include CASP1 (Proteintech, Catalog No. 22915-1-AP, concentration 1:300), NLRP 3 (ABWAYS TECHNOLOGY W, Catalog No. CY5651, concentration 1:100), AIM 2 (Bioss, Catalog No. bs-5986R, concentration 1:200), NLRP 1 (Proteintech, Catalog No. 12256-1-AP, concentration 1:300), and general SP kit (Beijing Zhongshan Jinqiao Biotechnology Co. Ltd., Catalog No. SP-9000). Others included EDTA antigen repair solution (Beijing Zhongshan Jinqiao Biotechnology Co. Ltd., Catalog No. ZLI-9067, concentration 1:49), DAB color development kit (Beijing Zhongshan Jinqiao Biotechnology Co. Ltd., Catalog No. ZLI-9018, substrate solution ratio: 1 mL to 50 *μ*L of concentrated DAB solution), and PBS (phosphate buffer solution) (Biosharp, Catalog No. BL601A).

#### 2.9.2. Immunohistochemistry Method

Immunohistochemical staining was performed using the EnVision two-step method. The tissues were embedded in paraffin, sectioned, and processed through steps including drying, antigen retrieval, antibody incubation, and DAB coloration. The dilution ratios for CASP1, NLRP3, AIM2, and NLRP1 were 1:300, 1:100, 1:200, and 1:300, respectively.

#### 2.9.3. Result Determination

Positive staining for CASP1, NLRP3, AIM2, and NLRP1 proteins appeared yellowish or brown, primarily localized in the cytoplasm or nucleus. The percentage of cell staining was graded on a scale of −0 to 12, with scores ranging from 0 to 6 and 7–12 points indicating low and high expressions, respectively. Low expression encompasses negative and weak positive staining, while high expression includes positive and strong positive staining [26]. The staining depth scores were categorized as negative (0 points), weakly positive (1 point), moderately positive (2 points), and strongly positive (3 points). The percentage fraction of cell staining was scored as follows: 0% (0 points), 1%–25% (1 point), 26%–50% (2 points), 50%–75% (3 points), and 76%–100% (4 points). A score of 1 point indicates a negative result, 2–3 points indicate a weak positive result, 4–6 points indicate a positive result, and a score greater than 6 points indicates a strongly positive result. Two doctors with intermediate or higher expertise levels at the Pathology Department of Hebei Province Chest Hospital, using a blind methodology, performed the assessment.

### 2.10. Statistical Analysis

Statistical analysis was performed using SPSS Version 19.0 software. Immunohistochemical staining data, which comprised counting data, was analyzed using the *χ*^2^ test. If the expected frequency was less than 5, the Fisher exact test was used. Operation process: weighted case; analysis → descriptive statistics → crosstabs → grouping →*χ*^2^ test. *p* < 0.05 was considered to be statistically significant.

## 3. Results

### 3.1. Screening for Pyroptosis Genes in Lung Adenocarcinoma

Enter “pyroptosis” into the search bar in the MSigDB, Gene, and GeneCards databases, respectively. The results from the MSigDB database showed 27 genes with the REACTOME_PYROPTOSIS category. Further exploration within the Gene database revealed 91 genes associated with pyroptosis. The GeneCards online database revealed 187 genes related to pyroptosis. Details can be found in Table 1.

Combining these three results, 202 genes related to pyroptosis were identified. These 202 genes were imported into the UALCAN database, revealing 127 DEGs upon analysis. Sixty-seven genes were upregulated, while 60 were downregulated in lung adenocarcinoma compared to normal tissues.  Table 2 shows the results for pyroptosis-related genes in lung adenocarcinoma.

### 3.2. Potential Functional Analysis of the DEGs

**Figure 2:**
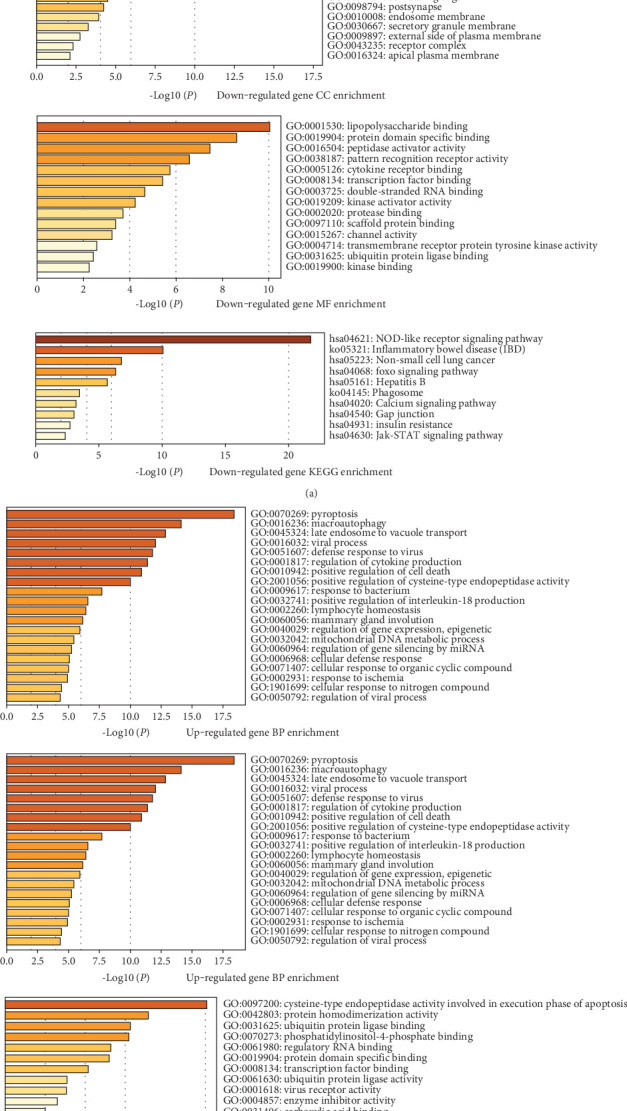
GO and KEGG enrichment analysis results. Functional enrichment results of (a) downregulated and (b) upregulated genes. Database: Metascape.

The Metascape database was employed to conduct DEG enrichment analysis. The upregulated and downregulated genes were input into the search bar. Parameter settings: input as species: any species; analysis as species: *H. sapiens* (5). Click submit analysis and the results are shown below. The outcome of GO enrichment centered on several critical pathways. These pathways included pyroptosis, defense response, cytokine and chemokine production regulation, response to lipopolysaccharide (LPS), apoptosis, phagocytosis, cellular response to mechanical stimuli, inflammasome assembly, and ESCRT III complex. Others included positive regulation of cysteine-type endopeptidase activity, Toll-like receptor signaling, NF-*κ*B signaling, and pattern recognition receptor binding. The KEGG enrichment results mainly focused on the NOD-like receptor signaling pathway, inflammatory bowel disease, non–small cell lung cancer (NSCLC), FOXO signaling pathway, JAK-STAT signaling pathway, cell apoptosis, and microbial infection.  Figure 2 shows the enrichment results.

### 3.3. PPI Network Map and Core Gene Selection

**Figure 3:**
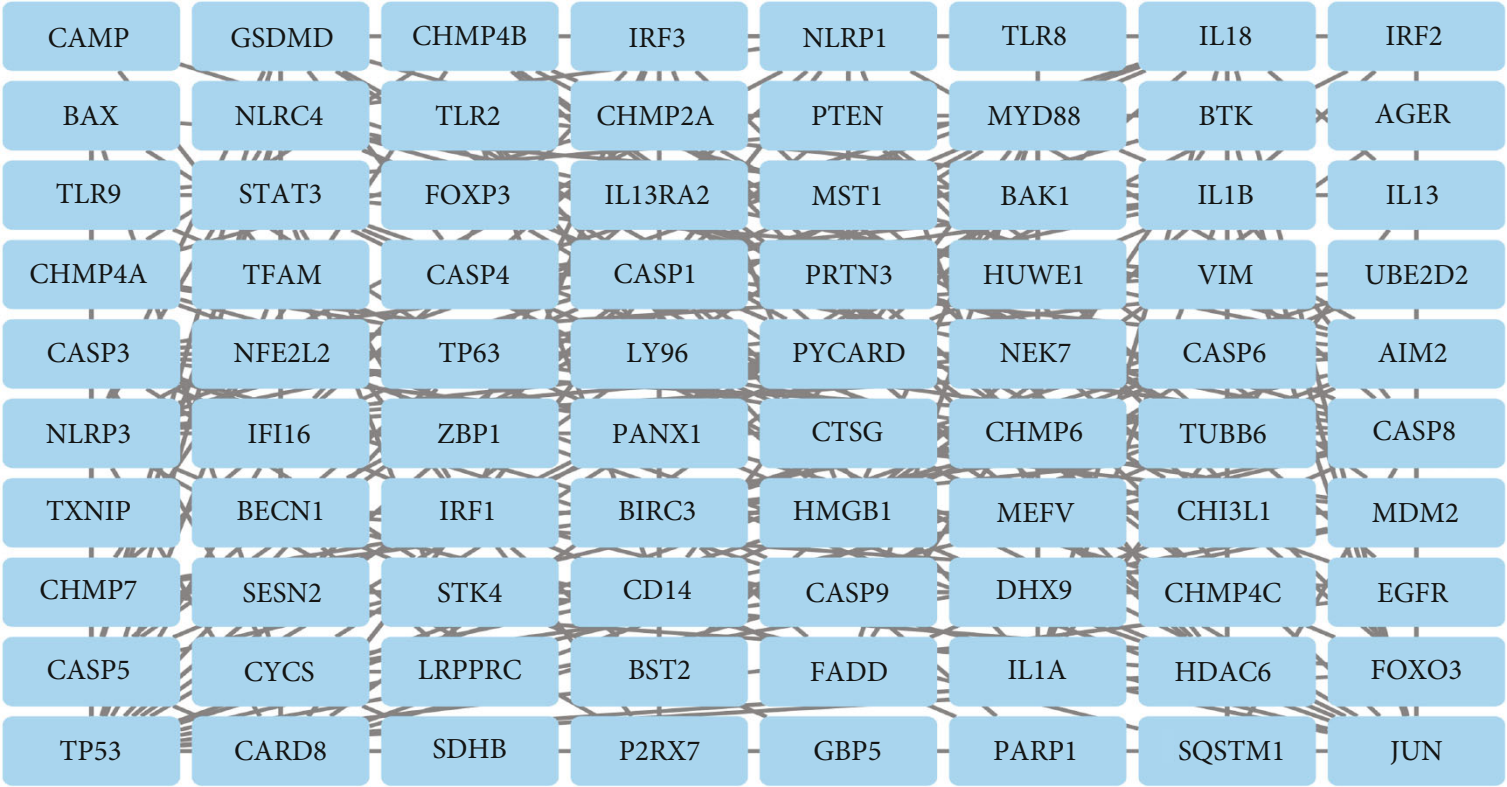
PPI. Database: STRING and Cytoscape software.

**Figure 4:**
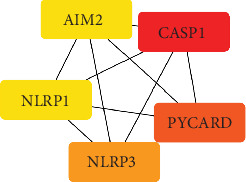
Core genes. Tool: cytoHubba.

The DEGs were entered into the STRING database, parameter settings: organisms: *H. sapiens*; network type: full STRING network; required score: high confidence (0.700); FDR stringency: medium (5%). And the data were visualized using the Cytoscape software, resulting in a PPI network map comprising 80 nodes and 228 edges.  Figure 3 shows the results. Moreover, the MCC algorithm in cytoHubba was utilized to select the top five core genes, namely, CASP1, PYCARD, NLRP3, AIM2, and NLRP1. The intensity of the red color denotes the significance the gene plays in biological processes.  Figure 4 shows the results.

### 3.4. Validation of Core Gene Expression

**Figure 5:**
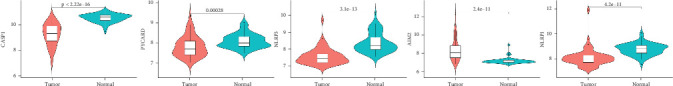
Verification of core gene differential expression. Database: GEO. Tool: jvenn.

CASP1, PYCARD, NLRP3, AIM2, and NLRP1 expression data were extracted from the GSE32863 dataset in the GEO database. These data were then input into the jvenn tool, revealing that CASP1, PYCARD, NLRP3, and NLRP1 exhibited low expression in lung adenocarcinoma compared to normal tissues. Additionally, the expression in normal tissues was consistent with the findings from the UALCAN database.  Figure 5 shows the results (lung adenocarcinoma tissue was the experimental group and normal tissue was the control group).

### 3.5. Correlation Between Immunization, Diagnostic Efficacy, and Prognosis

**Figure 6:**
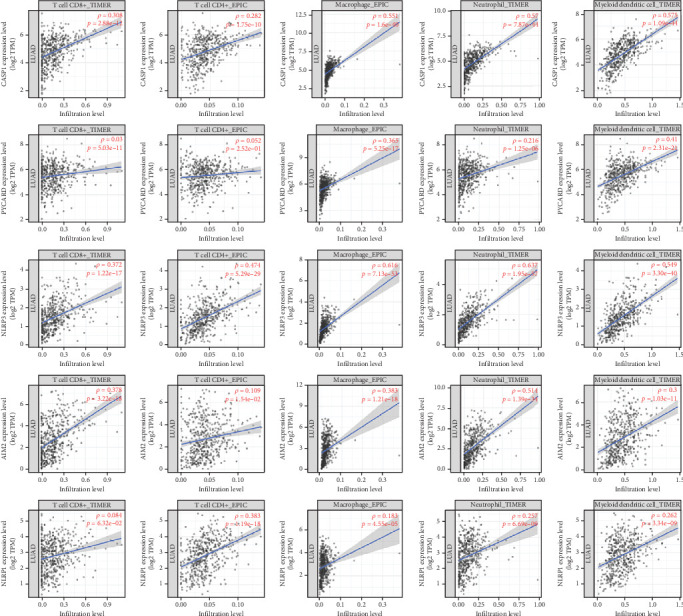
Correlation of immune infiltration. Database: TIMER.

**Figure 7:**
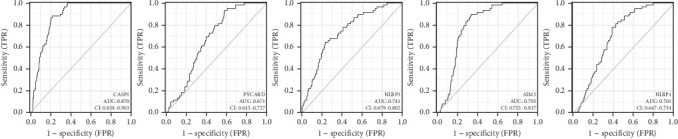
ROC diagnostic curves for CASP1, PYCARD, NLRP3, AIM2, and NLRP1. Database: TCGA. Tool: pROC package and ggplot2.

**Figure 8:**
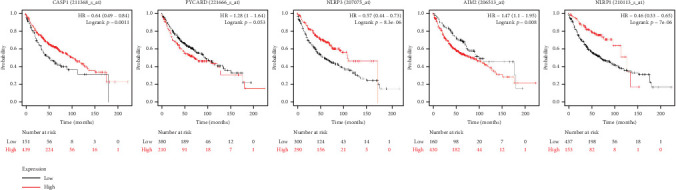
Survival analysis of CASP1, PYCARD, NLRP3, AIM2, and NLRP1. Database: Kaplan–Meier plotter.

Immunoinfiltration analysis was performed on CASP1, PYCARD, NLRP3, AIM2, and NLRP1 using the TIMER database, and the above genes were, respectively, input into the search bar. Parameter setting: lung cancer. The results from the TIMER database showed that CASP1, NLRP3, and AIM2 exhibited a positive correlation with the infiltration degree of CD8^+^ T cells, CD4^+^ T cells, macrophages, neutrophils, and dendritic cells. PYCARD was positively correlated with the infiltration degree of CD8^+^ T cells, macrophages, neutrophils, and dendritic cells, while no correlation was observed with CD4^+^ T cell infiltration. NLRP1 was positively correlated with the infiltration degree of CD4^+^ T cells, macrophages, neutrophils, and dendritic cells, while no correlation was observed in CD8^+^ T cell infiltration. The findings suggest an association between the core genes and immune cell infiltration in lung adenocarcinoma (Figure 6). ROC curves were generated to assess the potential efficiency of these five core genes as diagnostic biomarkers for lung adenocarcinoma. An area under the curve (AUC) > 0.700 indicates excellent diagnostic efficacy. The results showed the following AUC for each gene: CASP1 (AUC = 0.870, CI = 0.838–0.903), PYCARD (AUC = 0.671, CI = 0.615–0.727), NLRP3 (AUC = 0.741, CI =0.679–0.802), AIM2 (AUC = 0.795, CI = 0.752–0.837), and NLRP1 (AUC = 0.701, CI = 0.647–0.754). The results showed that CASP1, NLRP3, AIM2, and NLRP1 exhibited excellent diagnostic efficacy, suggesting their potential utility as diagnostic biomarkers for lung adenocarcinoma.  Figure 7 shows the results. The Kaplan–Meier plotter database results revealed a significant association between CASP1, NLRP3, AIM2, and NLRP1 expression and patient prognosis (*p* < 0.05). Furthermore, high CASP1, NLRP3, and NLRP1 expression showed favorable prognosis, while high AIM2 expression was associated with poor prognosis. However, PYCARD expression levels did not show a clear correlation with prognosis.  Figure 8 shows the results.

### 3.6. Build Predictive Nomogram

**Figure 9:**
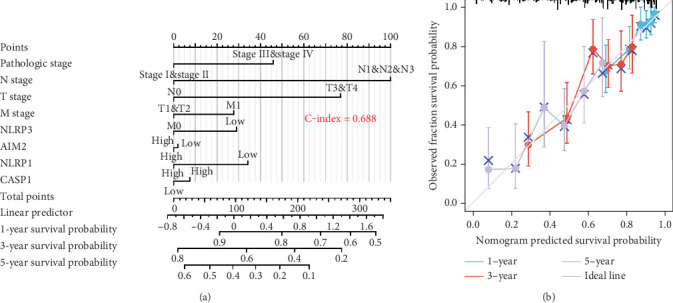
Prognostic model prediction. (a) Core gene's nomogram. (b) Nomogram's calibration diagram. Methods: R language. Tool: Cox regression.

To develop quantitative methods for predicting the prognosis of patients with lung adenocarcinoma, nomogram models were constructed using CASP1, NLRP3, AIM2, NLRP1, TNM, and pathological stage. The cumulative probability of OS was calculated by summing the assigned points corresponding to each variable in the nomogram model. The concordance index (*C*-index) of the nomogram model for predicting OS in patients with lung adenocarcinoma was 0.688. This index reflects the predictive ability of the model (Figure 9(a)). Calibration diagrams were created to predict 1-, 3-, and 5-year OS. The results showed a consistency between the predicted and actual OS, indicating a strong consistency between the predictive capacity of both models and observed outcomes (Figure 9(b)).

### 3.7. Correlation Between Nomogram Model and Immune Cell Infiltration

**Figure 10:**
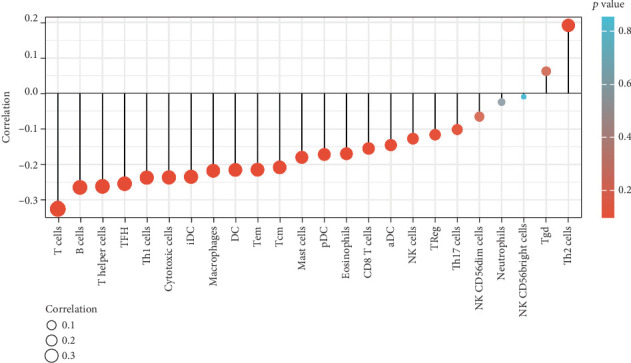
Correlation between nomograms and immune cell infiltration. Database: TCGA. Tool: R ggplot2.

Preliminary exploration using the TIMER database indicated a correlation between core genes and immune cells. Therefore, we examined whether this model exhibited an association with tumor immune infiltration. The findings revealed a correlation between the model and tumor-infiltrating immune cells. The risk score model demonstrated a negative correlation with immune cell infiltration, particularly T and B cells while showing a positive correlation with Tgd cells and Th2 cell infiltration (Figure 10).

### 3.8. Immunohistochemical Results of Core Genes

**Figure 11:**
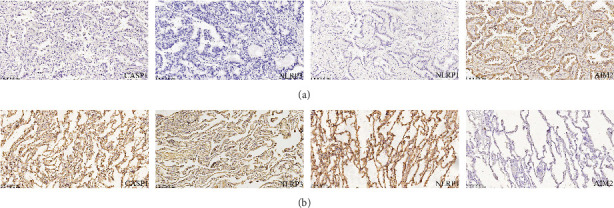
Expression of CASP1, NLRP3, NLRP1, and AIM2 in lung adenocarcinoma and adjacent tissues (SP 200). (a) Lung adenocarcinoma and (b) adjacent tissues.

CASP1, NLRP3, NLRP1, and AIM2 staining predominantly occurred in the cytoplasm. Immunohistochemical analysis are shown in Table 3. The findings demonstrated high expression of AIM2 in tumor tissues and low expression of CASP1, NLRP 3, and NLRP1 in tumor tissues. All observed differences were statistically significant (*p* < 0.05, Figure 11).

## 4. Discussion

We identified 127 DEGs in lung adenocarcinoma and normal tissues, suggesting their potential significance in developing and progressing lung adenocarcinoma. To explore the correlation between lung adenocarcinoma and pyroptosis, the DEGs were analyzed using the Metascape database. Our enrichment analysis findings suggest that these DEGs are involved in multiple pathways and stages related to pyroptosis. These pathways regulate cytokine production, phagocytosis, inflammasome assembly, positive regulation of cysteine-type endopeptidase activity, pattern recognition receptor binding, and response to LPSs. In the classical pyroptosis pathway, activation of pathogen-associated and damage-related molecular patterns leads to pro-CASP1 and PYCARD formation, which assemble to form inflammasomes. The inflammasome is a platform for activating the proteolytic enzyme CASP1, which can activate CASP1. CASP1 further plays a role in activating IL-1*β*, IL-18, and GSDMD. CASP1 cleaves GSDMD, resulting in the formation of N and C termini. The N-terminal part migrates to the cell membrane, forming pores that enable the cell membrane to rupture and facilitate the release of inflammatory factors [27]. In the nonclassical pyroptosis pathway, upon infecting the cells with Gram-negative bacilli, LPS directly induces the activation of CASP4, CASP5, and CASP11. These CASP family enzymes then cleave GSDMD to induce pyroptosis [28]. Moreover, these enrichment findings are intricately linked to lung adenocarcinomas. They involve processes such as regulating cytokine production, inflammasome assembly, NF-*κ*B signaling pathway, FOXO, and JAK-STAT signaling pathways. Cytokines influence lung cancer progression and can serve as diagnostic and prognostic biomarkers. Cytokines promote or limit tumor growth by regulating critically related signaling pathways such as proliferation, growth, invasion, differentiation, migration, metastasis, and apoptosis. Studies by Tan et al. [29] and Laird et al. [30] highlight the association of cytokine IL-1*β* with growth, migration, invasion, and poor prognosis of lung adenocarcinoma. The NLRP3 inflammasome enhances lung adenocarcinoma cell A549 proliferation by activating the IL-1*β*/ERK/CREB and IL-18/AKT/CREK signaling pathways [31]. The NF-*κ*B signaling pathway is known to be activated in lung cancer. Targeting NF-*κ*B has demonstrated the ability to inhibit cell viability, stimulate G2/M phase arrest, promote apoptosis, inhibit tumor growth, and induce apoptosis [32]. FOXO is involved in various cellular functions, including cell differentiation, apoptosis, cell proliferation, and DNA damage and repair. Moreover, it is considered to play a key role as a tumor suppressor in numerous cancer types [33]. JAK-STAT regulates cell development, differentiation, proliferation, and apoptosis. It participates in normal physiological process regulation and is crucial in the occurrence and development of tumors. JAK-STAT 1 has been confirmed to promote the apoptosis of lung cancer cells [34]. These studies suggest that these DEGs not only participate in the occurrence and development of pyroptosis but also participate in the pyroptosis of lung cancer cells through various signaling pathways.

The top five core genes associated with pyroptosis, including CASP1, PYCARD, NLRP3, AIM2, and NLRP1, were analyzed using the STRING database and Cytoscape software. These core genes predominantly participate in the classical and nonclassical pyroptosis pathways. In the classical pyroptosis pathway, a key step is the formation of the inflammasome—a multimeric complex structure composing cytoplasmic sensors, apoptosis-associated spot-like protein containing a CASP activation domain with PYCARD, and pro-CASP1 [35]. Various forms of inflammasomes have been identified in previous studies, including NLRP1, NLRC4, AIM2, NLRP3, and pyrin inflammasomes [11]. Once assembled, these inflammasomes can activate the CASP family, inducing the occurrence of cell pyroptosis. The initial step in the nonclassical pyroptosis pathway is the cellular response to LPS. Upon cell infection with Gram-negative bacilli, LPS directly activates the CASP family, inducing the nonclassical pyroptosis pathway [2]. These findings suggest the pivotal involvement of core genes in pyroptosis development and their close association with lung adenocarcinoma. Zhang et al. demonstrated a positive correlation between p53 expression levels and NLRP3, PYCARD, and CASP1 mRNA levels in NSCLC [36]. *In vitro*, p53 directly modulates pyroptosis in lung adenocarcinoma A549 cells. *In vivo*, p53 overexpression significantly reduces tumor growth and the mortality rate of xenograft tumors in a nude mouse model [36]. Qi et al. found that AIM2 promotes NSCLC development by regulating mitochondrial dynamics [37]. Studies on NLRP1 have predominantly concentrated on nonneoplastic lung diseases. No evidence exists to clarify the precise association between NLRP1 and lung adenocarcinoma.

Immune regulation is crucial in lung adenocarcinoma progression. The number and proportion of infiltrating immune cells are pivotal factors affecting cancer progression and response to immunotherapy. They are associated with patient prognosis [38]. Therefore, obtaining a comprehensive understanding of immune cell infiltration patterns within the pyroptosis-mediated tumor microenvironment is crucial. This understanding can advance our knowledge of tumor immunity and aid in facilitating immunotherapeutic strategies, considering factors such as inflammatory factor secretion and immune responses. Several studies have found that GSDME boosts tumor-associated macrophage phagocytosis and increases the number and efficacy of tumor-infiltrating NK cells and CD8^+^ T lymphocytes [39]. We analyzed the immune correlation of pivotal genes concerning lung adenocarcinoma and demonstrated their association with the infiltration of immune cells, including CD8^+^ T lymphocytes. Therefore, a risk model was established to explore the relationship between the risk model and tumor-infiltrating immune cells. The findings revealed a negative association between the risk model and T and B cell infiltration. Conversely, it exhibited a positive correlation with Tgd and Th2 cells. This further confirms the pyroptosis core gene CASP1, NLRP3, AIM2, and NLRP1 expression levels in lung adenocarcinoma and their connection to immune infiltration. These findings provide a novel perspective for clinical immunotherapy.

We aim to identify lung adenocarcinoma markers to facilitate early diagnosis for improved therapeutic outcomes. Evaluation using ROC curves for core genes indicated that CASP1, NLRP3, AIM2, and NLRP1 demonstrate excellent diagnostic potential for lung adenocarcinoma and may serve as diagnostic markers for the disease. However, PYCARD exhibited poor diagnostic efficacy; hence, it is not recommended as a diagnostic marker of lung adenocarcinoma. We further analyzed the influence of core genes on lung adenocarcinoma prognosis, and survival analysis revealed a significant association between CASP1, NLRP3, NLRP1, and AIM2 and overall survival in patients with lung adenocarcinoma. Nonetheless, PYCARD did not show any association with the prognosis of lung adenocarcinoma in patients. Based on the diagnostic and prognostic evaluation of core genes, PYCARD was excluded from consideration. Subsequently, CASP1, NLRP3, NLRP1, and AIM2 expression levels were integrated with TNM and pathological stages to comprehensively evaluate a nomogram. The nomogram effectively quantifies and visualizes the results derived from Cox regression analysis, providing an intuitive prediction of patient prognosis. Previous studies have confirmed the utility of nomogram models in predicting the prognosis of patients with various tumors, including breast cancer [25]. In this study, the nomogram model was established, which was then validated using the calibration curve. The results demonstrated a strong association between the nomogram prediction model and actual OS. This association will help in screening patients with poor prognoses and identifying the population that could benefit the most.

The treatment of lung cancer has developed from chemotherapy and radiotherapy to immunotherapy and targeted therapy. Chemotherapy, radiotherapy, immunotherapy, and targeted therapy can induce pyroptosis in tumors, suggesting that pyroptosis may broaden the therapeutic field of tumors, enhance clinical outcomes, and improve clinical prognosis in the near future [40]. Chemotherapy can cause pyroptosis of lung cancer cells. Zhang et al. found that both paclitaxel and cisplatin significantly induced the death of lung adenocarcinoma A549 cells, and these dead cells showed the characteristics of pyroptosis [41]. Pyroptosis is associated with immunotherapy of lung cancer. Lu et al. designed a novel chimeric costimulatory converting receptor (CCCR) to interfere with PD1 signaling and enhance the activity of CAR-NK cells against solid tumors [42]. It mainly includes the extracellular domain of PD1, the transmembrane and intracellular segment of NKG2D, and the intracellular segment of costimulatory molecule 41BB. CCCR-NK92 cells were able to convert PD1 inhibitory signals into activation signals, thereby reversing the immunosuppressive effects of PD1, and showed enhanced antitumor activity against human lung cancer H1299 cells in vitro; the rapid death of which was caused by pyroptosis induced by CCCR-NK92 cells [42]. Targeted therapy can cause pyroptosis of lung cancer cells. In order to analyze the correlation between targeted therapy and pyroptosis of lung cancer, Lu et al. use western blot analysis, phase contrast imaging, scanning electron microscope, and flow cytometry to analyze the cell death models of A549 (KRAS-G12S mutation), PC9 (EGFR19 exon deletion mutation), and NCI-H3122 (EML4-ALK fusion gene) lung cancer cell lines and xenotransplantation models after targeted therapy. The results show that lung cancer cells not only experienced apoptosis but also pyroptosis [43]. At present, there is no direct report on the correlation between radiotherapy and pyroptosis of lung cancer, but the correlation between radiotherapy and pyroptosis of colorectal cancer has been confirmed. Tan et al. find that GSDME caused radiation-induced pyroptosis in colorectal cancer cells and normal epithelial cells through the CASP3-dependent pathway [44]. The expression of GSDME makes radiation-resistant colorectal cancer cells sensitive to radiation [44].

To verify the credibility of the findings from the analysis, 20 paraffin-embedded pathological specimens of surgically resected lung adenocarcinomas were selected for immunohistochemical testing. The results showed elevated levels of AIM2 in tumor tissues, while CASP1, NLRP3, and NLRP1 exhibited low expression in these tumor tissues. The immunohistochemical examination of the core genes in lung adenocarcinoma confirmed the correlation between lung adenocarcinoma occurrence and pyroptosis, as indicated in the biocredit analysis.

Our focus was constructing a diagnostic and prognostic model for lung adenocarcinoma based on the core genes—CASP1, NLRP3, AIM2, and NLRP1. This model holds significant potential for enhancing the clinical diagnosis and treatment strategy for patients with lung adenocarcinoma. These findings shed light on the mechanism underlying lung adenocarcinoma development and have significant implications for identifying potential therapeutic targets and prognostic molecular markers.

This study has inherent limitations. Our investigation focused solely on the role of apoptosis-related genes in lung adenocarcinoma, which is influenced by various factors, The research was limited to immunohistochemical validation, lacking in-depth exploration of specific molecular mechanisms. Software algorithms and sequencing systems also have limitations. Our next goal is to validate the diagnostic and prognostic value of these apoptosis-related genes in lung adenocarcinoma in larger patient cohorts and diverse populations.

## 5. Conclusion

CASP1, NLRP3, AIM2, and NLRP1 are core pyroptotic genes in lung adenocarcinoma, significantly associated with immune cell infiltration, diagnosis, and prognosis in this condition. Their relevance suggests potential clinical utility in diagnosing and treating patients with lung adenocarcinoma.

## Figures and Tables

**Table 1:** The GeneCards online database revealed 187 genes related to pyroptosis.

MSigDB database	BAX, CHMP2B, GZMB, ELANE, GSDMD, CHMP6, CASP1, IL1B, IL1A, CHMP4C, GSDME, CASP3, CASP4, CHMP2A, CHMP7, CHMP4B, CHMP3, BAK1, CYCS, HMGB1, BTK, MDM2, SIRT1, BIRC3, SCAF11, BECN1, P2RX7
Gene database	TP53, IL1B, TNF, APOE, NLRP3, STAT3, NFKB1, VDR, PTEN, CD274, IL18, HMGB1, CASP1, CASP8, NFE2L2, CASP3, DRD2, AGER, GZMB, GJA1, MIR155, TREM2, ELANE, FOXO3, ABL1, BSG, MALAT1, VIM, AIM2, NLRP1, ADORA2A, CAMP, DHX9, CEBPB, FGF21, GSDMD, MIR223, BRD4, PECAM1, GSDME, CDKN2B-AS1, MEG3, CASP4, SDHB, MRE11, MIR214, IL32, TXNIP, TFAM, MIR204, MIR22, PYCARD, CAPN1, PRDM1, ATF6, GSDMB, GZMA, NR1H2, NLRC4, ADORA1, MIR497, KCNQ1OT1, UTS2, NAIP, ADORA2B, METTL3, ADORA3, MIR25, NLRP7, CARD8, EEF2K, ZBP1, MIR30C1, IL36G, NLRP6, MIR485, CTSV, GSDMA, TUBB6, GSDMC, NEK7, APIP, TRIM31, CRTAC1, DLX6-AS1, NLRP9, ZDHHC1, PYDC2, KLF3-AS1, NEWENTRY, PTGS2
GeneCards online database	GSDMD, GSDME, NLRP3, CASP1, GSDMB, CASP4, GSDMC, NLRP1, GSDMA, CARD8, GZMB, IL1B, GZMA, DPP9, DPP8, NLRC4, CASP8, CASP5, AIM2, ZBP1, PYCARD, NAIP, DHX9, NLRP9, CASP3, IL18, HMGB1, APIP, KCNQ1OT1, MIR223, TREM2, FOXO3, MALAT1, CASP6, TXNIP, MIR22, DDX3X, MIR125A, GBP1, GJA1, MIR30C1, PRDM1, MIR214, UBR2, CPTP, TP53, VDR, BRD4, NEK7, CRTAC1, NFE2L2, AGER, TET2, UTS2, CTSV, MIR155, NFKB1, APOE, SDHB, EEF2K, P2RX7, CD274, FGF21, KLF3-AS1, CEBPB, TFAM, BSG, IL32, MEG3, MIR21, MIR135B, MIR485, MALT1, STK4, MST1, PRF1, ELAVL1, CDKN2B-AS1, MIR204, MIR9-1, MIR9-3, MIR9-2, MIR497, HDAC6, SQSTM1, IRF3, ZDHHC1, STING1, HNP1, PTEN, ADORA1, ADORA2B, ADORA3, ADORA2A, METTL3, PECAM1, TRIM31, MIR25, CAMP, MRE11, PARP1, GBP5, NR1H2, CTSG, MKI67, IL36G, IL36B, PRTN3, SERPINB1, NLRP6, APOL1, FOXP3, NLRP7, BNIP3, ANO6, XIST, MIR103A2, MIR103A1, FADD, SESN2, TNF, VIM, CAPN1, JUN, MIR139, MEFV, ALK, SIRT1, BIRC3, BIRC2, UBE2D2, RIPK3, LY96, GLMN, IRGM, SCAF11, NLRP13, ADAMTS9-AS2, TUBB6, MYD88, TLR8, NOS2, NOS1, PYDC2, ACE2, AKT1, EGFR, CASP9, TP63, ATF6, IRF1, IFI16, IRF2, POP1, ORMDL3, BTK, MDM2, STAT3, BCL2, TLR2, ANXA2, IL1RN, BECN1, CD14, GSTO1, IL13, HUWE1, TLR9, TNFSF13B, ASIC1, CHI3L1, PANX1, LRPPRC, CXCL8, IL13RA2, BST2, GPER1, LYST, NCR1, CLEC5A, CGAS, GAS5, MIR15A, MIR20B

**Table 2:** Pyroptosis-related genes in lung adenocarcinoma.

**Type**	**Gene**
Up	ACE2, ADORA2B, ADORA3, AIM2, ATF6, BAK1, BAX, BECN1, BIRC3, BNIP3, BSG, BST2, CAPN1, CASP3, CASP4, CASP6, CASP8, CASP9, CHI3L1, CHMP2A, CHMP4A, CHMP4B, CHMP4C, CHMP6, CHMP7, CLEC5A, CYCS, DPP9, DHX9, ELAVL1, FADD, FOXP3, GAS5, GBP5, GLMN, GSDMA, GSDMB, GSDMC, GSDMD, HDAC6, HUWE1, IFI16, IRF3, KCNQ1OT1, LRPPRC, LY96, MALAT1, MEG3, METTL3, MKI67, MST1, NLRP7, PANX1, PARP1, POP1, SDHB, SESN2, SQSTM1, TFAM, TLR9, TP53, TRIM31, UBE2D2, UBR2, UTS2, VDR, ZBP1
Down	ADORA1, ADORA2A, AGER, ALK, ANO6, ANXA2, APIP, APOL1, BTK, CAMP, CARD8, CASP1, CASP5, CD14, CD274, CRTAC1, CTSG, DDX3X, EGFR, FOXO3, GJA1, GSTO1, HMGB1, IL1A, IL1B, IL13, IL13RA2, IL18, IRF1, IRF2, JUN, MDM2, MEFV, MYD88, NCR1, NEK7, NFE2L2, NLRC4, NLRP1, NLRP3, NOS1, NR1H2, ORMDL3, P2RX7, PECAM1, PRF1, PRTN3, PTEN, PYCARD, STAT3, STK4, TLR2, TLR8, TP63, TREM2, TUBB6, TXNIP, VIM, XIST, ZDHHC1

**Table 3:** The results of immunohistochemistry.

	**CASP1**		**NLRP3**		**NLRP1**		**AIM2**	
	**Positive (** **n** **)**	**Negative (** **n** **)**	**Positive (** **n** **)**	**Negative (** **n** **)**	**Positive (** **n** **)**	**Negative (** **n** **)**	**Positive (** **n** **)**	**Negative (** **n** **)**
Lung adenocarcinoma (*n*)	4	16	3	17	2	18	14	6
Adjacent tissues (*n*)	6	4	6	4	7	3	2	8
*p*	0.045		0.03		0.002		0.019	

## Data Availability

The datasets used and/or analyzed during the current study are available from the corresponding authors on reasonable request.

## References

[1] Sung H., Ferlay J., Siegel R. L. (2021). Global cancer statistics 2020: GLOBOCAN estimates of incidence and mortality worldwide for 36 cancers in 185 countries. *CA: A Cancer Journal for Clinicians*.

[2] Yang F., Bettadapura S. N., Smeltzer M. S., Zhu H., Wang S. (2022). Pyroptosis and pyroptosis-inducing cancer drugs. *Acta Pharmacologica Sinica*.

[3] Wang W. J., Chen D., Jiang M. Z. (2018). Downregulation of gasdermin D promotes gastric cancer proliferation by regulating cell cycle-related proteins. *Journal of Digestive Diseases*.

[4] Barber G., Anand A., Oficjalska K. (2020). Characterizing caspase-1 involvement during esophageal disease progression. *Cancer Immunology, Immunotherapy*.

[5] Zhang L., Zeyu W., Liu B., Jang S., Zhang Z., Jiang Y. (2021). Pyroptosis in liver disease. *Revista Española de Enfermedades Digestivas*.

[6] Berkel C., Cacan E. (2021). Differential expression and copy number variation of gasdermin (GSDM) family members, pore-forming proteins in pyroptosis, in normal and malignant serous ovarian tissue. *Inflammation*.

[7] Zhou C. B., Fang J. Y. (2019). The role of pyroptosis in gastrointestinal cancer and immune responses to intestinal microbial infection. *Biochimica Et Biophysica Acta Reviews on Cancer*.

[8] Chen X., Chen H., Yao H. (2021). Turning up the heat on non-immunoreactive tumors: pyroptosis influences the tumor immune microenvironment in bladder cancer. *Oncogene*.

[9] Liu W., Peng J., Xiao M. (2023). The implication of pyroptosis in cancer immunology: current advances and prospects. *Genes & Diseases*.

[10] Watanabe K., Stringer S., Frei O. (2019). A global overview of pleiotropy and genetic architecture in complex traits. *Nature Genetics*.

[11] Fang Y., Tian S., Pan Y. (2020). Pyroptosis: a new frontier in cancer. *Biomedicine & Pharmacotherapy*.

[12] Liberzon A., Birger C., Thorvaldsdóttir H., Ghandi M., Mesirov J. P., Tamayo P. (2015). The molecular signatures database (MSigDB) hallmark gene set collection. *Cell Systems*.

[13] Brown G. R., Hem V., Katz K. S. (2015). Gene: a gene-centered information resource at NCBI. *Nucleic Acids Research*.

[14] Safran M., Dalah I., Alexander J. (2010). GeneCards version 3: the human gene integrator. *Database*.

[15] Chandrashekar D. S., Bashel B., Balasubramanya S. A. H. (2017). UALCAN: a portal for facilitating tumor subgroup gene expression and survival analyses. *Neoplasia*.

[16] Zhou Y., Zhou B., Pache L. (2019). Metascape provides a biologist-oriented resource for the analysis of systems-level datasets. *Nature Communications*.

[17] Szklarczyk D., Gable A. L., Lyon D. (2019). STRING v11: protein-protein association networks with increased coverage, supporting functional discovery in genome-wide experimental datasets. *Nucleic Acids Research*.

[18] Shannon P., Markiel A., Ozier O. (2003). Cytoscape: a software environment for integrated models of biomolecular interaction networks. *Genome Research*.

[19] Chin C. H., Chen S. H., Wu H. H., Ho C. W., Ko M. T., Lin C. Y. (2014). cytoHubba: identifying hub objects and sub-networks from complex interactome. *BMC Systems Biology*.

[20] Barrett T., Wilhite S. E., Ledoux P. (2012). NCBI GEO: archive for functional genomics data sets—update. *Nucleic Acids Research*.

[21] Bardou P., Mariette J., Escudié F., Djemiel C., Klopp C. (2014). jvenn: an interactive Venn diagram viewer. *BMC Bioinformatics*.

[22] Li T., Fu J., Zeng Z. (2020). TIMER2.0 for analysis of tumor-infiltrating immune cells. *Nucleic Acids Research*.

[23] Liu J., Lichtenberg T., Hoadley K. A. (2018). An integrated TCGA pan-cancer clinical data resource to drive high-quality survival outcome analytics. *Cell*.

[24] Nagy Á., Lánczky A., Menyhárt O., Győrffy B. (2018). Validation of miRNA prognostic power in hepatocellular carcinoma using expression data of independent datasets. *Scientific Reports*.

[25] Park S. Y. (2018). Nomogram: an analogue tool to deliver digital knowledge. *The Journal of Thoracic and Cardiovascular Surgery*.

[26] Yang Y. C., Cheng T. Y., Huang S. M. (2017). Cytosolic PKM2 stabilizes mutant EGFR protein expression through regulating HSP90-EGFR association. *Oncogene*.

[27] Yu P., Zhang X., Liu N., Tang L., Peng C., Chen X. (2021). Pyroptosis: mechanisms and diseases. *Signal Transduction and Targeted Therapy*.

[28] Kim H. M., Kim Y. M. (2018). HMGB1: LPS delivery vehicle for caspase-11-mediated pyroptosis. *Immunity*.

[29] Tan Q., Duan L., Huang Q. (2021). Interleukin-1*β* promotes lung adenocarcinoma growth and invasion through promoting glycolysis via p38 pathway. *Journal of Inflammation Research*.

[30] Laird B. J., McMillan D., Skipworth R. J. E. (2021). The emerging role of interleukin 1*β* (IL-1*β*) in cancer cachexia. *Inflammation*.

[31] Wang Y., Kong H., Zeng X. (2016). Activation of NLRP3 inflammasome enhances the proliferation and migration of A549 lung cancer cells. *Oncology Reports*.

[32] Chen L., Li Q., Zheng Z. (2019). Design and optimize N-substituted EF24 as effective and low toxicity NF-*κ*B inhibitor for lung cancer therapy via apoptosis-to-pyroptosis switch. *Chemical Biology & Drug Design*.

[33] Wang B., Wang Z. M., Ji J. L. (2020). Macrophage-derived exosomal Mir-155 regulating cardiomyocyte pyroptosis and hypertrophy in uremic cardiomyopathy. *Basic to Translational Science*.

[34] Pastuszak-Lewandoska D., Domańska-Senderowska D., Kordiak J. (2017). Immunoexpression analysis of selected JAK/STAT pathway molecules in patients with non-small-cell lung cancer. *Polish Archives of Internal Medicine*.

[35] Chi K., Geng X., Liu C., Cai G., Hong Q. (2020). Research progress on the role of inflammasomes in kidney disease. *Mediators of Inflammation*.

[36] Zhang T., Li Y., Zhu R. (2019). Transcription factor p53 suppresses tumor growth by prompting pyroptosis in non-small-cell lung cancer. *Oxidative Medicine and Cellular Longevity*.

[37] Qi M., Dai D., Liu J. (2020). AIM2 promotes the development of non-small cell lung cancer by modulating mitochondrial dynamics. *Oncogene*.

[38] Yao J., Chen X., Liu X., Li R., Zhou X., Qu Y. (2021). Characterization of a ferroptosis and iron-metabolism related lncRNA signature in lung adenocarcinoma. *Cancer Cell International*.

[39] Zhang Z., Zhang Y., Xia S. (2020). Gasdermin E suppresses tumour growth by activating anti-tumour immunity. *Nature*.

[40] Tan Y., Chen Q., Li X. (2021). Pyroptosis: a new paradigm of cell death for fighting against cancer. *Journal of Experimental & Clinical Cancer Research*.

[41] Zhang C. C., Li C. G., Wang Y. F. (2019). Chemotherapeutic paclitaxel and cisplatin differentially induce pyroptosis in A549 lung cancer cells via caspase-3/GSDME activation. *Apoptosis*.

[42] Lu C., Guo C., Chen H. (2020). A novel chimeric PD1-NKG2D-41BB receptor enhances antitumor activity of NK92 cells against human lung cancer H1299 cells by triggering pyroptosis. *Molecular Immunology*.

[43] Lu H., Zhang S., Wu J. (2018). Molecular targeted therapies elicit concurrent apoptotic and GSDME-dependent pyroptotic tumor cell death. *Clinical Cancer Research*.

[44] Tan G., Lin C., Huang C. (2022). Radiosensitivity of colorectal cancer and radiation-induced gut damages are regulated by gasdermin E. *Cancer Letters*.

